# High Frequency Mutations in *pfdhfr* and *pfdhps* of *Plasmodium falciparum* in Response to Sulfadoxine-Pyrimethamine: A Cross-Sectional Survey in Returning Chinese Migrants From Africa

**DOI:** 10.3389/fcimb.2021.673194

**Published:** 2021-09-08

**Authors:** He Yan, Jun Feng, Jian-hai Yin, Fang Huang, Xiang-li Kong, Kang-ming Lin, Tao Zhang, Xin-yu Feng, Shui-sen Zhou, Jian-ping Cao, Zhi-gui Xia

**Affiliations:** ^1^National Institute of Parasitic Diseases, Chinese Center for Disease Control and Prevention (Chinese Center for Tropical Diseases Research), NHC Key Laboratory of Parasite and Vector Biology, WHO Collaborating Centre for Tropical Diseases, National Center for International Research on Tropical Diseases, Shanghai, China; ^2^School of Global Health, Chinese Center for Tropical Diseases Research, Shanghai Jiao Tong University School of Medicine, Shanghai, China; ^3^Shandong Institute of Parasitic Diseases, Shandong First Medical University & Shandong Academy of Medical Sciences, Shandong, China; ^4^Instit of Parasitic Diseases, Guangxi Zhuang Autonomous Region Center for Disease Control and Prevention, Guangxi, China; ^5^Anhu Provincial Center for Disease Control and Prevention, Anhui, China

**Keywords:** *Plasmodium falciparum*, sulfadoxine-pyrimethamine, dihydropteroate synthase, dihydrofolate reductase, migrants from Africa

## Abstract

**Background:**

Sulfadoxine-pyrimethamine (SP) is recommended for intermittent preventive treatment in Africa against *Plasmodium falciparum* infection. However, increasing SP resistance (SPR) of *P. falciparum* affects the therapeutic efficacy of SP, and *pfdhfr* (encoding dihydrofolate reductase) and *pfdhps* (encoding dihydropteroate synthase) genes are widely used as molecular markers for SPR surveillance. In the present study, we analyzed single nucleotide polymorphisms (SNPs) of *pfdhfr* and *pfdhps* in *P. falciparum* isolated from infected Chinese migrant workers returning from Africa.

**Methods:**

In total, 159 blood samples from *P. falciparum*-infected workers who had returned from Africa to Anhui, Shangdong, and Guangxi provinces were successfully detected and analyzed from 2017 to 2019. The SNPs in *pfdhfr* and *pfdhps* were analyzed using nested PCR. The genotypes and linkage disequilibrium (LD) were analyzed using Haploview.

**Results:**

High frequencies of the Asn51Ile (N51I), Cys59Arg(C59R), and Ser108Asn(S108N) mutant alleles were observed, with mutation frequencies of 97.60, 87.43, and 97.01% in *pfdhfr*, respectively. A triple mutation (IRN) in *pfdhfr* was the most prevalent haplotype (86.83%). Six point mutations were detected in *pfdhps* DNA fragment, Ile431Val (I431V), Ser436Ala (S436A), Ala437Gly (A437G), Lys540Glu(K540E), Ala581Gly(A581G), Ala613Ser(A613S). The *pfdhps* K540E (27.67%) was the most predominant allele, followed by S436A (27.04%), and a single mutant haplotype (SGKAA; 62.66%) was predominant in *pfdhps*. In total, 5 haplotypes of the *pfdhfr* gene and 13 haplotypes of the *pfdhps* gene were identified. A total of 130 isolates with 12 unique haplotypes were found in the *pfdhfr-pfdhps* combined haplotypes, most of them (n = 85, 65.38%) carried quadruple allele combinations (CIRNI-SGKAA).

**Conclusion:**

A high prevalence of point mutations in the *pfdhfr* and *pfdhps* genes of *P. falciparum* isolates was detected among Chinese migrant workers returning from Africa. Therefore, continuous *in vitro* molecular monitoring of Sulfadoxine-Pyrimethemine combined *in vivo* therapeutic monitoring of artemisinin combination therapy (ACT) efficacy and additional control efforts among migrant workers are urgently needed.

## Introduction

Imported *Plasmodium falciparum* infections are of great concern in China during malaria elimination and post-elimination stages, because China achieved zero indigenous malaria cases in 2017 (Feng et al., 2018). Appropriate treatment for *P. falciparum* according to the national plan is important; however, some studies have demonstrated mutations in molecular markers related to artemisinin combination therapy (ACT) resistance among isolates from Africa, suggesting that careful surveillance of African parasite populations is still warranted (Feng et al., 2015a; Feng et al., 2019, Yan et al., 2020).

Sulfadoxine-pyrimethamine (SP) is a second-line antimalarial for uncomplicated *P. falciparum* malaria treatment, as recommended by the World Health Organization (WHO), which is used as an intermittent preventive treatment in pregnancy (IPTp) and as an intermittent preventive treatment in infants (IPTi) in malaria-endemic regions (Gosling et al., 2010; Konate et al., 2011). Resistance to SP is caused mainly by point mutations in *pfdhfr* (encoding dihydrofolate reductase) and *pfdhps* (encoding dihydropteroate synthase). Mutations in *pfdhfr* and *pfdhps* have been associated with decreased parasite sensitivity to the SP, because the products of these genes could incrementally increase the parasite’s tolerance to the drugs *in vitro* (Chulay et al. 1984). Studies have identified point mutations in codons N51I, C59R, and S108N, I164L of *pfdhfr* located on chromosome 4 and codons I431V, S436A, A437G, K540E, A581G, and A613S of *pfdhps* located on chromosome 8, all of which were associated with *P. falciparum* SP treatment failure (Triglia et al., 1997; Berglez et al., 2004; Pearce et al., 2009). Monitoring drug resistance and the pattern of mutations is essential for early detection and subsequent prevention of the spread of drug resistance. The present study identified the polymorphisms in *pfdhfr* and *pfdhps* in *P. falciparum* among returned migrant workers from Africa in 2017–2019 reported in eastern China. The results provide a deeper understanding of the disease as well as baseline information on antimalarial drug resistance among imported *P. falciparum* in China.

## Materials and Methods

### Sample Sites

#### Sample Collection and DNA Extraction

The study was conducted in Anhui, Shandong, and Guangxi Provinces in Eastern and Southern China, where imported *P. falciparum* cases were predominantly. Anhui Province covers 105 counties with 70.6 million people and experienced a malaria resurgence in 2005–2008 that was mainly caused by the accumulation of residual foci of *P. vivax* (Feng et al., 2015b). Shandong Province, located in eastern China, has a long coastline measuring 3,024.4 kilometers. It contains 137 counties and has a population of 97.9 million. Economic trade overseas is frequent. Another province, Guangxi, is known for its gold miners who returned from Ghana in 2013, which was mainly reported in Shanglin County (Feng et al., 2015a). The number of imported *P. falciparum* cases, especially those from Africa, has increased significantly in these three provinces, and 360 P*. falciparum* cases were reported in 2019, accounting for 18.5% of all *P. falciparum* cases nationwide (Zhang L et al., 2020).

**Figure 1:**
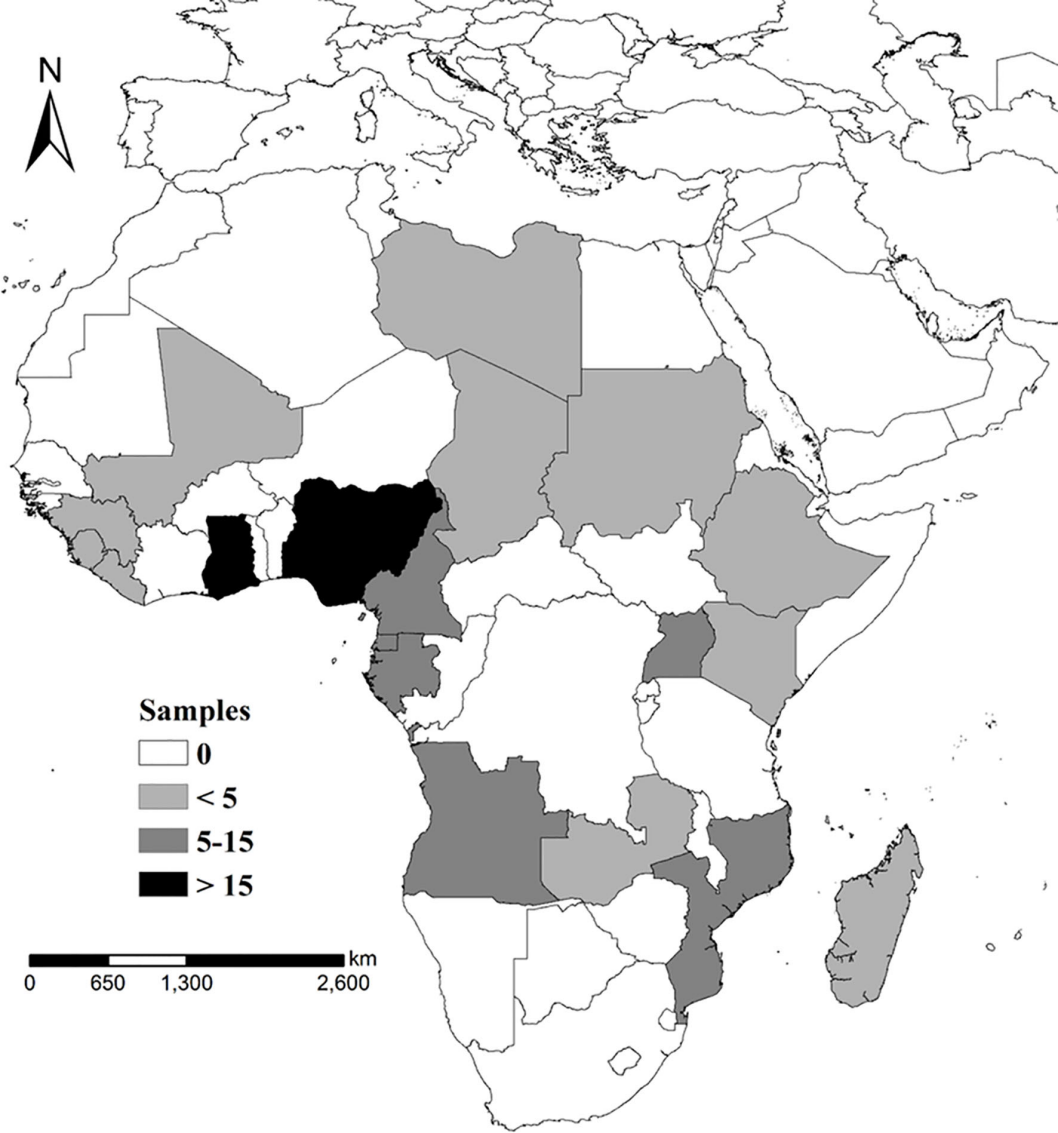
Sample collection and distribution, samples collection sites and sample screening and follow-up analysis of subject patterns.

A total of 206 *P. falciparum*-infected blood samples were collected from the travelers returning from Africa from 2017 to 2019. The samples distribution were shown as **Figure 1**. Approximately 100 μl of finger-prick blood was spotted onto a piece of 3MM Whatman filter paper (GE Healthcare, Boston, MA, USA) and air dried.

The *Plasmodium falciparum* genomic DNA from the approximately 100 μl of collected blood sample was extracted using a QIAamp DNA blood kit (QIAGEN, Valencia, CA, USA) as described previously (Yan et al., 2020). Each of the samples was labeled with a study number and stored at −4°C until extraction. Individual epidemiological information was also collected using a web-based reporting system (China Information System for Diseases Control and Prevention) and analyzed.

### Detection of *pfdhfr* and *pfdhps* Polymorphisms

Point mutations at codons 16, 51, 59, 108, and 164 of the *pfdhfr* gene and codons 431, 436, 437, 540, 581, and 613 of the *pfdhps* gene were evaluated using nested PCR amplification. The sequences of the primers used for *pfdhfr* and *pfdhps* genotyping were as described previously (Zhang et al., 2014). The primary amplification was performed using the following parameters: 1 cycle of 95°C for 3 min; 35 cycles of 95°C for 30 s, 55°C for 30 s, and 65°C for 6 for 30 s, and 1 cycle of 65°C for 60 s; and 65°C for 5 min. The second amplifcation was performed using the following parameters: 1 cycle of 95°C for 3 min; 35 cycles of 95°C for 30 s, 52°C for 30 s, and 65°C for 60 s; and 1 cycle of 65°C for 5 min. 750-bp product of *pfdhps* were sent for Sanger sequencing (Shanghai Bunan Biological Co., Ltd., Shanghai, China).

### Data Analysis

Sequences were analyzed using the Blast program (http://blast.ncbi.nlm.nih.gov/). Multiple nucleotide sequence alignments and analysis were carried out using the MAFFT web-based tool with the Cluster Omega Sequence Alignment Editor (https://www.ebi.ac.uk/Tools/msa/clustalo/). Sequences with poor quality after three sequencing attempts or those with more than one peak at one locus were not included in the analysis. The map showing the imported of countries with number of the isolates was created by ArcGIS 10.1 (Environmental Systems Research Institute, Inc., Redlands, CA, USA). SPSS18.0 (IBM Corp., Armonk, NY, USA) was used to conduct the statistical analyses, and the Chi-squared test was employed to test the different constituent ratios of *pfdhfr* and *pfdhps* gene polymorphisms. The Fisher’s precision probability test was used as the sample size is less 50; For the sample size less than five would be discarded when analyzing the geographical distribution difference. The inter and intragenic SNP Linkage disequilibrium (LD) associations of *pfdhfr* and *pfdhps* were analyzed using Haploview 15 (Patel et al., 2017).

### Ethical Considerations

This study was reviewed and approved by the ethical committee of the National Institute of Parasitic Diseases, Chinese Centre for Disease Control and Prevention (NIPD, China CDC, No. 2019008).

## Results

### Epidemiological Information

Among the 206 *P. falciparum* isolates collected in this study, 202 and 177 isolates were successfully amplified and sequenced for the *pfdhfr* and *pfdhps* genes, respectively; however, only single infections and both of *pfdhfr* and *pfdhps* successful amplicons were involved in the final analysis. Therefore, a total of 159 P*. falciparum* isolates were successfully detected and analyzed in this study (**Figure 1**). Their distribution was identified as 2 from North Africa, 23 from East Africa, 54 from West Africa, and 80 from Central Africa. Among them, the Democratic Republic of the Congo (n = 28), Nigeria (n = 27), and Angola (n = 16) were considered as the top three imported source countries. The average patient age was 42 years, and 154 patients (154/159, 96.86%) were male. The numbers of cases reported in 2017, 2018, and 2019 were 28, 58, and 73, respectively.

### Prevalence of *pfdhfr* Polymorphisms

For *pfdhfr*, no polymorphism was found for codons 50 or 164. Compared with mutations N51I and C59R, S108N had a higher SNP prevalence, whereas, the difference between N51I and C59R was not significant. Only three isolates were sequenced as wild-type Asparagine-Cysteine-Serine (NCS) (accounting for 1.89%), whereas, the triple-mutant genotype, Isoleucine-Arginine-Asparagine **(**IRN), comprised 80.50% (n = 128), the others were NCN (n = 3, 1.89%), ICN (n = 10, 6.29%), and NRN (n = 15, 9.43%), respectively (**Table 1**).

**Table 1:** Prevalence of *pfdhfr* and *pfdhps* polymorphisms in imported African isolates.

Gene	Position	Wild type	Mutant type	Mutant isolates(n)	Frequency(%)
*pfdhfr*	51	AAT	ATT	138	86.79
	59	TGT	**C**GT	143	89.94
	108	AGC	A**A**C	156	98.11
*pfdhps*	431	ATA	**G**TA	12	7.55
	436	TCT	**G**CT	43	27.04
	437	GCT	G**G**T	15	9.43
	540	AAA	**G**AA	44	27.67
	581	GCG	G**G**G	14	8.81
	613	GCC	**T**CC	3	1.89

The bold values stand for mutant nucleic acid base.

### Prevalence of *pfdhps* Polymorphisms

The SNPs of *pfdhps* were relatively scattered, S436A (27.07%) and K540E (27.67%) carried a higher allele frequency, which were statistically significant than others (the average mutant frequency was 6.92%). The frequencies of the other three alleles, I431V, A437G, and A581G, were lower than those of S436A or K540E, but higher than that of S613A, which was carried by only two isolates. In all, among 159 P*. falciparum* isolates, 13 kinds of mutants of *pfdhps* were detected. Further sequencing showed that 73 single mutant isolates, including ISA**E**AA (the bold residue represents the mutated site; n = 41, 25.79%), I**A**AKAA (n = 24, 15.09%), IS**G**KAA (n = 6, 3.77%), and ISAK**G**A (n = 2, 1.26%); 13 double mutant isolates, including I**AG**KAA (n = 7, 4.40%), **VA**AKAA (n = 2, 1.26%), ISA**EG**A (n = 2, 1.26%), I**A**AKA**S** (n = 1, 0.63%), and I**A**A**E**AA (n = 1, 0.63%); 9 triple mutant genotypes, comprising 8 examples of **VA**AK**G**A, 1 of **V**S**G**K**G**A; and only 1 quadruple mutation, as **VA**AK**GS,** were identified. There were more wild-type isolates of *pfdhps* than p*fdhfr* (63 *pfdhps* isolates, compared with three in *pfdhfr*). Three genotypes, ISAKAA (39.62%, 63/159), ISA**E**AA (25.79%, 41/159), and I**A**AKAA (15.09%, 24/159), accounted for 80.50% of all *pfdhps* genotypes (**Table 2**).

**Table 2:** Prevalence of *pfdhfr* and *pfdhps* haplotypes.

Gene	Genotype	Mutations	Sample size	Frequency (%)
*pfdhfr*	NCS	0	3	1.89%
	NC**N**	1	3	1.89%
	**I**C**N**	2	10	6.29%
	N**RN**	2	15	9.43%
	**IRN**	3	128	80.50%
*pfdhps*	ISAKAA	0	63	39.62%
	ISA**E**AA	1	41	25.79%
	I**A**AKAA	1	24	15.09%
	IS**G**KAA	1	6	3.77%
	ISAK**G**A	1	2	1.26%
	**VA**AKAA	2	2	1.26%
	I**AG**KAA	2	7	4.40%
	ISA**EG**A	2	2	1.26%
	I**A**AKA**S**	2	1	0.63%
	I**A**A**E**AA	2	1	0.63%
	**VA**AK**G**A	3	8	5.03%
	**V**S**G**K**G**A	3	1	0.63%
	**VA**AK**GS**	4	1	0.63%

The bold values stand for mutant nucleic acid base.

### Geographical Genetic Analysis

The mutant frequency in all targeted *pfdhfr* gene fragments were all above 86.79%, and there was no significant difference (P > 0.05) among isolates from West, East, and Central Africa. For the *pfdhps* gene, the mutant genotypes carried a relatively high number of polymorphisms. The two *P. falciparum* samples from North Africa were wild-types (**Table 3**). Two of six loci were detected as site mutations in samples from East Africa, with K540E representing 69.57% (16/23) of the mutations, which was a markedly higher frequency than that for S436A (4.34%, 1/23). Each locus carried a sense mutation associated with West Africa, the mutant frequencies were 14.81% (8/54, I413V), 51.85% (28/54, S436V), 9.26% (5/54, A437G), 11.11% (6/54, K540E), 12.96% (7/54, A581G), and 5.56% (3/54, A613S). The site mutations in central African region were all different for the S436V (17.5%, 14/80, P < 0.001) and K540E (27.5%, 22/80, P = 0.017) loci, which was significantly different compared with the Western African Region isolates (P < 0.05). Moreover, the mutant frequency of K540E varied between Western and Eastern African Region isolates (P <0.01). A613S only occurred in Ghana (n = 2) and Nigeria (n = 1) in West Africa.

**Table 3:** Geographical genetic analysis of *pfdhfr/pfdhps* genotypes.

Region	No. samples	Country	No. samples	No. of mutations	*pfdhfr/pfdhps* genotypes	No. of genotypes
North Africa	2	Libya	1	3	IRN/ISAKAA	1
		Sudan	1	3	IRN/ISAKAA	1
East Africa	23	Kenya	1	3	IRN/ISAKAA	1
		Madagascar	2	2	NRN/ISAKAA	2
		Ethiopia	3	3	IRN/ISAKAA	1
				4	IRN/IAAKAA	1
				4	IRN/ISAEAA	1
		Zambia	4	3	NRN/ISAEAA	1
				4	IRN/ISAEAA	3
		Uganda	6	4	IRN/ISAEAA	6
		Mozambique	7	2	ICN/ISAKAA	1
				4	IRN/ISAEAA	6
West Africa	54	Mali	1	3	IRN/ISAKAA	1
		Sierra Leone	2	2	NCN/IAAKAA	1
				3	IRN/ISAKAA	1
		Guinea	2	3	IRN/ISAKAA	1
				4	IRN/ISGKAA	1
		Liberia	3	1	NCN/ISAKAA	1
				4	IRN/IAAKAA	2
		Cote d'lvoire	8	0	NCS/ISAKAA	1
				2	NRN/ISAKAA	1
				3	IRN/ISAKAA	2
				4	IRN/IAAKAA	3
				4	IRN/ISAEAA	1
		Ghana	16	1	NCS/IAAKAA	1
				2	NRN/ISAKAA	1
				3	IRN/ISAKAA	3
				3	ICN/ISAEAA	1
				3	NRN/IAAKAA	1
				4	IRN/IAAKAA	3
				4	IRN/ISAEAA	2
				4	ICN/IAGKAA	1
				4	NRN/IAAKAS	1
				5	IRN/IAAEAA	1
				5	IRN/IAGKAA	1
		Nigeria	22	2	NRN/ISAKAA	1
				3	IRN/ISAKAA	6
				4	IRN/IAAKAA	5
				4	IRN/ISAEAA	1
				5	IRN/IAGKAA	1
				5	IRN/VAAKAA	1
				5	NRN/VAAKGS	1
				6	IRN/VAAKGA	6
Central Africa	80	Chad	1	6	IRN/VSGKGA	1
		Central Africa	3	2	NRN/ISAKAA	2
				3	IRN/ISAKAA	1
		Congo	4	2	NRN/ISAKAA	2
				3	IRN/ISAKAA	2
		Equatorial Guinea	6	3	IRN/ISAKAA	3
				4	IRN/ISAKGA	1
				5	IRN/ISAEGA	1
				6	IRN/VAAKGA	1
		Gabon	10	3	IRN/ISAKAA	7
				4	IRN/ISAEAA	3
		Angola	14	2	ICN/ISAKAA	2
				2	NCN/ISGKAA	1
				3	IRN/ISAKAA	4
				4	IRN/IAAKAA	2
				4	IRN/ISAKGA	1
				4	IRN/ISAEAA	3
				4	IRN/ISGKAA	1
		Cameroon	14	3	IRN/ISAKAA	5
				4	IRN/IAAKAA	4
				4	IRN/ISAEAA	1
				5	IRN/IAGKAA	2
				5	IRN/VAAKAA	1
				6	IRN/VAAKGA	1
		DR Congo	28	2	ICN/ISAKAA	1
				2	NRN/ISAKAA	1
				3	IRN/ISAKAA	6
				3	NRN/ISAEAA	1
				3	ICN/ISAEAA	3
				4	IRN/IAAKAA	1
				4	IRN/ISGKAA	3
				4	ICN/ISAEGA	1
				4	IRN/ISAEAA	9
				5	IRN/IAGKAA	2

### Linkage Disequilibrium (LD) Analysis

**Figure 2:**
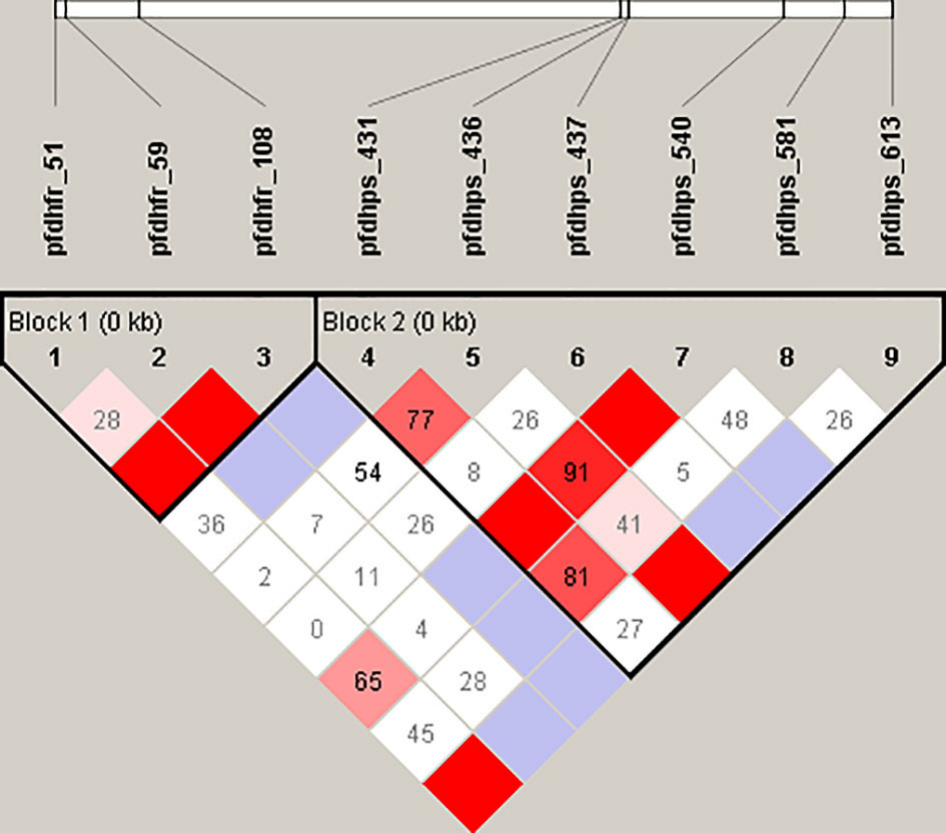
Linkage disequilibrium (LD) analysis of *pfdhfr* and *pfdhps* SNPs. For the *pfdhfr* gene, the single amino acid mutations were N51I, C59R, and S108N, respectively. Likewise, *pfdhps* gene are related to the mutations S436A, A437G, K540E, and A613S, respectively. The dark and light red squares indicate a linkage that was statistically significant (P < 0.05). Cambridge blue squares indicate a linkage that is present but is not statistically significant (P > 0.05). A white square with indicates that no linkage is present.

In total, 25 genotypes of *pfdhfr/pfdhps* were detected among the 159 P*. falciparum* isolates. For *pfdhfr*, no SNP was found for codons 16 or 164. Ultimately, 5 and 13 genotypes of *pfdhfr* (involving codons 51, 59, and 108) and *pfdhps* (involving codons 431, 436, 437, 540, 581, and 613) were detected and analyzed. To evaluate the SNP LD associations of *pfdhfr* and *pfdhps*, several statistically significant associations were found among the SNPs located in both the *pfdhfr* and *pfdhps* genes (**Figure 2**). For the *pfdhfr* gene, N51I was significantly associated with the SNPs (t175c, C59R; and g323a, S108N) with a D’ value of 0.84 (P < 0.05) and 1.0 (P < 0.05), respectively. Similarly, t175c was significantly associated with the g323a (0.71, P < 0.05). For the *pfdhps* gene, t1482g, c1486g, a1794g, and g2013t formed an LD block. The sole SNP (t1482g, S436A) was significantly associated with the SNPs (c1486g, A437G; a1794g, K540E; and g2013t, A613S) with D’ values of 0.75, 1.0, and 0.73, respectively. The SNP (t152a) of the *pfdhfr* gene encoding N51I was significantly associated with c1486g, with a D’ value of 0.68. No associations were detected for the other SNPs of either the *pfdhfr* or *pfdhps* genes.

## Discussion

The emergence and spread of Sulfadoxine-Pyrimethamine (SP) resistance has narrowed its usage to IPTp and IPTi in Africa (Rieckmann and Cheng, 2002). In 2019, the WHO covered 33 African countries for IPTp, in which at least nearly 62% of pregnant women received a first dose of IPTp (of four doses of ITPp)) (World Health Organization, 2020). The presents study aimed to determine the prevalence of SP resistance-associated *pfdhfr* and *pfdhps* genes in *P. falciparum* isolated from returned migrant workers from Africa reported in China. The study showed the *pfdhfr* triple haplotype mutation (**IRN**, including N51I, C59R, S108N) was highly prevalent, which was similar to other publications concerning African isolates (Jiang et al., 2019; Gikunju et al., 2020; Quan et al., 2020). This high prevalence was also found in the migrant workers returning to Guangxi from Ghana, suggesting that SP resistant genotype was widespread in Central and West Africa (Zhao et al., 2020). It was noted *pfdhfr* I164L, which was also associated with high resistance to cycloguanil, was detected in Ghanaian isolates, but was not found in our study (Zhao et al., 2020). It is worth noting that I164L is common in East Africa and Asia (Basuki et al., 2018,; Lynch et al., 2017), but rarely seen in Central and West Africa. One of the explanations was that this mutation site carries a high fitness cost to the parasite and therefore it is unable to survive the immune response of hosts in West Africa (Nzila et al., 2002). Further studies are needed to assess the effect of this mutation on the phenotype of parasites carrying this haplotype.

Mutations in *pfdhps* haplotypes at S436A and K540E, which are associated with decreased parasite sensitivity to Sulfadoxine drugs (Berglez et al., 2004), carried a higher allele frequency, which was also found in Uganda and Tanzania (Alifrangis et al., 2009; Mbonye et al., 2015). The WHO recommended that IPTp should not implemented in the regions when K540E exceed 50%; in our study, this mutation was present at 27.67%, which might favor the continued efficacy of IPTp treatment in these countries. Another mutation, A581G, is considered to have an important modulatory role in SP resistance. IPTp and SP could not protect pregnant women from delivering low birth weight infants when the frequency of this mutation is above 10% (Chico et al., 2015). In our study, the frequency of this mutation was 8.81% and half of these mutated isolates were found in Nigeria. For the mutation A437G, which was associated with resistance to Sulfadoxine in endemic regions because of the drug pressure selection, showed a frequency of only 9.43% in our study, which was lower than that reported in Equatorial Guinea, Pakistan, and Iran (Rouhani et al., 2015; Yaqoob et al., 2018; Jiang et al., 2019). The I431V mutation was detected in Nigeria, Cameroon, Equatorial Guinea, and Chad. The most frequent haplotype of I431V was **VA**AK**G**A, similar to that found in isolates from Cameroon and Nigeria (Chauvin et al., 2015; Oguike et al., 2016). These haplotypes occurring in Central and West Africa suggested that SP conferred a selective advantage, and ongoing drug pressure is relative strong because SP was used as IPTp in these regions. It is noted A437G was widely spread in central and western African countries, which indicating *in vivo* SP drug resistance in these regions (Pearce et al., 2009). Some other studies combined with the clinical study indicated the *pfdhfr* triple mutant genotype was associated with SP treatment resistance (Basco et al, 2000; Naidoo et al., 2013). However, in this study, the A437G was not widely spread in central and western African region, even less than S436A, which was also different with our previous study in China-Myanmar border (Zhang et al., 2014), which may partially ascribe as the sample size limitation, the loss of drug pressure, and time passage may also the potential reason.

In our study, the frequency of the quadruple mutation **IRN**/ISA**E**AA was significantly higher in East Africa (100% in Uganda, 85.7% in Mozambique) than western and central African countries (P < 0.01), suggesting that the clinical implications of such haplotypes require further combined genotype and phenotype analysis. SP was limited for malaria control among the general population in many countries in Africa because of the high frequency of drug resistance developed by *P. falciparum*; therefore, we expected to obtain SP sensitive strains. Indeed, in our study, 39.62% of the isolates harbored the non-mutated *pfdhps* gene. This may be similar to the process for chloroquine, which tends to recover its effectiveness against the parasite after a long period during which its use in malaria control activities is halted (Lu et al., 2017).

## Conclusion

This study showed a high frequency of SP-resistance associated SNPs in the *pfdhfr* and *pfdhps* genes of *P. falciparum* isolated since 2017 in returned migrant workers from Africa. The high resistance may be linked to the unsuccessful withdrawal of the SP treatment, and thus might affect the efficacy of IPTp for pregnant women and IPTi for infants. Mutations such as K540E, and the *pfdhfr-pfdhps* haplotype **IRN**/ISA**E**AA, which occurred at moderate frequencies in East Africa, such as in Uganda and Mozambique, the other regions showed as high frequency triple mutations of *pfdhfr*, but relatively scatter site mutations in *pfdhps* gene. The present data could provide the evidence for molecular surveillance in the post-elimination stage in China, focusing on the risk population among returning migrant workers from Africa, and could be used to determine the treatment policy for imported malaria in China.

## Data Availability Statement

The raw data supporting the conclusions of this article will be made available by the authors, without undue reservation.

## Ethics Statement

The studies involving human participants were reviewed and approved by the ethical committee of the National Institute of Parasitic Diseases (NIPD), Chinese Centre for Disease Control and Prevention, Chinese Center for Tropical Diseases Research. The patients/participants provided their written informed consent to participate in this study.

## Author Contributions

HY conceived the study. J-hY, FH, X-yF, and JF performed the study and analyzed the data. JF assisted with editing the manuscript. J-pC provided strategic advice and revised the manuscript. All authors assisted with draft revisions. All authors contributed to the article and approved the submitted version.

## Funding

The work was supported by the Key Techniques in Collaborative Prevention and Control of Major Infectious Diseases in the Belt and Road (Grant No. 2018ZX10101002-004) and the National Natural Science Foundation of China (Grant No. 81602904). The study had also been supported by “the Fifth Round of Three-Year Public Health Action Plan of Shanghai (No. GWV-10.1-XK13)”.

## Conflict of Interest

The authors declare that the research was conducted in the absence of any commercial or financial relationships that could be construed as a potential conflict of interest.
